# Design of the DIRECT-project: interventions to increase job resources and recovery opportunities to improve job-related health, well-being, and performance outcomes in nursing homes

**DOI:** 10.1186/1471-2458-10-293

**Published:** 2010-05-28

**Authors:** Ellen Spoor, Jan de Jonge, Jan PH Hamers

**Affiliations:** 1 Department of Industrial Engineering & Innovation Sciences, Human Performance Management Group, Eindhoven University of Technology, Eindhoven, the Netherlands; 2 CAPHRI school for Public Health and Primary Care, Department of Health Care and Nursing Science, Maastricht University, Maastricht, the Netherlands

## Abstract

**Background:**

Because of high demands at work, nurses are at high risk for occupational burnout and physical complaints. The presence of job resources (such as job autonomy or social support) and recovery opportunities could counteract the adverse effect of high job demands. However, it is still unclear how job resources and recovery opportunities can be translated into effective workplace interventions aiming to improve employee health, well-being, and performance-related outcomes. The aim of the current research project is developing and implementing interventions to optimize job resources and recovery opportunities, which may lead to improved health, well-being and performance of nurses.

**Methods/design:**

The DIRECT-project (DIsc Risk Evaluating Controlled Trial) is a longitudinal, quasi-experimental field study. Nursing home staff of 4 intervention wards and 4 comparison wards will be involved. Based on the results of a base-line survey, interventions will be implemented to optimize job resources and recovery opportunities. After 12 and 24 month the effect of the interventions will be investigated with follow-up surveys. Additionally, a process evaluation will be conducted to map factors that either stimulated or hindered successful implementation as well as the effectiveness of the interventions.

**Discussion:**

The DIRECT-project fulfils a strong need for intervention research in the field of work, stress, performance, and health. The results could reveal (1) how interventions can be tailored to optimize job resources and recovery opportunities, in order to counteract job demands, and (2) what the effects of these interventions will be on health, well-being, and performance of nursing staff.

## Background

Nurses are at high risk for occupational burnout [1-3] and physical complaints, merely due to high demands at work [4,5]. Because job demands often cannot be easily reduced, it is rather appealing to counteract the adverse effects of high job demands on well-being and health with so-called job resources [6]. Job resources can be broadly conceptualized as different kinds of energey reservoirs that can be tapped when the employee has to cope with job demands [7,8]. Examples of job resources are job autonomy, colleagues providing help, sympathy, and affection, and ergonomic aids. Empirical research has shown that job resources are important stress buffers for nurses [9]. In addition, Taris et al. [10] showed that a well-balanced mix of job demands and job resources is positive related to well-being, and that well-being is positive related to performance indicators such as client satisfaction and efficiency.

Besides the availability of job resources to counteract high job demands, recovery from job demands can also be an important prerequisite for not feeling strained and for performing well when returning to work the next day. According to Meijman and Mulder [11], recovery occurs when no further demands are put on those aspects of an individual's functioning on which job demands have been put during the work process. There is empirical evidence showing that recovery from work is positively related to employees' health and well-being [12-15], as well as to job performance [16].

Because job demands can often not be reduced, organizations need to implement workplace interventions that revolve around the management of job resources and recovery opportunities. However, there is a gap between theoretical knowledge gained from work stress and performance models and their practical implications [17]. According to Kompier and Taris [18], there is a strong need for intervention research in the field of work, stress, performance, and health. The current research project fills-in this gap by developing and implementing interventions to optimize job resources and recovery opportunities. This may lead to improved health and performance of nurses. Because a systematic analysis of risk factors is often lacking in stress intervention research [19], a strength of the present study is the adequate diagnosis of risk factors conform a recent developed theoretical model called the Demand-Induced Strain Compensation (DISC) Model [6,20]. This is in line with Goldenhar et al. [21], who called for *theory-driven *intervention research to learn why and under what circumstances work-oriented interventions succeed.

In more detail, the DISC Model treats job demands and job resources as multidimensional constructs. In line with Hockey [22], three type of job demands can be distinguished: (1) cognitive demands which primarily impinge on the brain processes involved in information processing [22], (2) emotional demands which primarily refer to the effort needed to deal with organizationally desired emotions during interpersonal transactions [23], and (3) physical demands which are primarily associated with the musculo-skeletal system [22]. Similarly, job resources may encompass a cognitive component (e.g. access to useful information from books), an emotional component (e.g. colleagues providing sympathy and affection), and a physical component (e.g. ergonomic aids). Finally, in line with Sonnentag and Niessen [24], recovery from work can also be divided in a cognitive component (think on other things than work), an emotional component (put all emotions from work aside), and a physical component (i.e. not longer affected by the work posture). Based upon homeostatic functional self-regulation processes, the DISC Model proposes that job resources as well as recovery within the same domain as the job demands (i.e. cognitive, emotional, or physical) will produce a greater likelihood of counteracting the negative job demands and creates optimal conditions for health, well-being, and performance [25]. For example, high emotional job demands (e.g. dealing with dying patients) can lead to emotional exhaustion, unless employees have high emotional resources (e.g. social support from colleagues) and a high level of emotional recovery (e.g. meeting with friends after work) to counteract the high emotional demands.

### Aim and research question

The aim of the current study is developing, implementing, and evaluating interventions to optimize job resources and recovery opportunities, which may lead to improved health, well-being and performance of nurses. The interventions that will be implemented after a baseline measure, will be based on the central principle of the DISC Model, i.e. the amount of specific job resources and recovery opportunities have to match with the corresponding types of job demands to optimize job-related outcomes. These interventions will be primarily work-oriented, i.e. changing the work situation rather than changing the employee or his/her perceptions. A meta-analysis of stress management interventions [26] showed that only a few studies assessed the impact of work-oriented interventions, and that there is a paucity of research which compared different kinds of interventions on respective, similar, outcomes. The research question is: Does a tailored intervention program based upon the DISC Model result in an improvement of employee health, well-being and performance-related outcomes? In other words, how can job demands, job resources, and recovery both during and after work time be optimized to improve health, well-being and performance of nursing staff?

## Methods and design

### Overview

**Figure 1:**
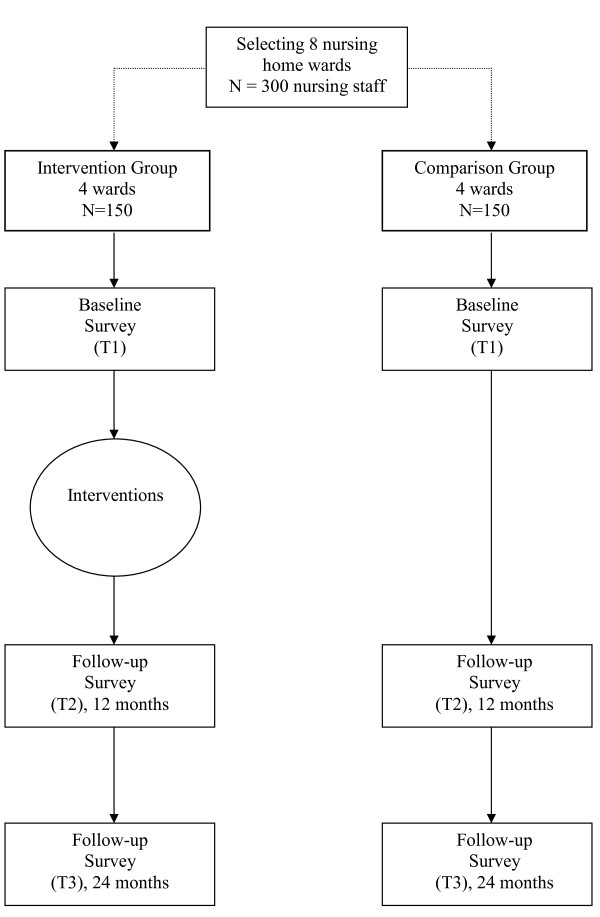
**Flow chart design and measurements**.

The DIRECT-project (DIsc Risk Evaluating Controlled Trial) is a longitudinal, quasi-experimental field study (February 2009-February 2011) with a 'non-equivalent control group pretest posttest design' [27]. Employees within two Dutch nursing homes will participate in the research project, one in the North of the Netherlands and one in the South. In the nursing home in the North of the Netherlands two wards on one location will be the intervention group and two wards on another location will be the comparison group. In the Southern nursing home two wards will be the intervention group and two wards in the same location will be the comparison group. In total, four wards will be the intervention group (*n *= 150) and four wards will be the comparison group (*n *= 150). The more realistic terminology 'comparison group' was selected explicitly in contrast to 'control group' as the formal requirements for a real control group are not met [28]. Figure 1 presents a flow-chart of the design and measurements. Based on the result of the base-line survey (T1), workplace interventions will be implemented to optimize job demands, job resources, and recovery both during and after work time. One year after the baseline measurement, the results of the interventions will be evaluated by a follow-up survey (T2). To investigate if the interventions lead to enduring higher performance, well-being and employee health, a second follow-up survey will be conducted two year after the base-line measure (T3). In addition to the follow-up surveys, which will measure the effect of the interventions, a process evaluation will be performed.

### Participants

All nursing staff (i.e. nursing assistants, certified nursing assistants, and registered nurses) working on a permanent basis at one of the eight wards, will be eligible to participate in the study. Because of the longitudinal character of the project, temporary staff will be excluded.

### Measures

The measures that will be used in both the baseline survey and the follow-up surveys are described below.

### DISC-Model measures

*Cognitive, emotional and physical job demands and job resources *will be measured with a well-validated version of the DISC Questionnaire (DISQ), which was particularly developed for testing this theoretical model [9,29]. *Cognitive job demands *primarily impinge on brain processes involved in information processing, e.g., "Employee X will need to display high levels of concentration and precision at work". *Emotional job demands *can be defined as the effort needed to deal with job inherent emotions and/or organizationally desired emotions during interpersonal transactions, e.g., "Employee X will have to display emotions (e.g., towards clients, colleagues or supervisors) that are inconsistent with his/her current feelings". *Physical job demands *refer to static and dynamic physical exertion at work, e.g., "Employee X will have to lift or move heavy persons or objects (more than 10 kg)". *Cognitive job resources *refer to the opportunity to determine a variety of task aspects and to use problem solving skills, e.g., "Employee X would have the opportunity to take a break when tasks require a lot of concentration". To improve the internal reliability of the scale, one item was added to the original questionnaire ("Employee X will have the opportunity to determine their own work method"). *Emotional job resources *refer to emotional support from colleagues or supervisors, e.g., "Other people (e.g., clients, colleagues or supervisors) would be a listening ear for employee X when he/she has faced a threatening situation". Finally, *physical job resources *refer to instrumental support from colleagues and supervisors, or ergonomic aids at work, e.g., "Employee X would receive help from others (e.g., clients, colleagues or supervisors) in lifting or moving heavy persons or objects". All but two scales consist of five items (except for emotional demands and cognitive resources: six items) that can be scored on a 5-point frequency scale, ranging from 1 *(never or very rarely) *to 5 *(very often or always) Recovery from work*. Recovery refers to an employee's sense of being away from the work situation. It will be measured with a scale developed by De Jonge et. al. ("Take a break?" Off-job recovery, job demands and job resources as predictors of active learning, creativity, and health, submitted) and may encompass a cognitive, emotional and physical component. Each component will be measured with three items. Example items are: "After work, I put all thoughts of work aside" (cognitive), "After work, I emotionally distance myself from work" (emotional), "After work, I shake off the physical exertion from work" (physical). The items can be scored on a 5-point frequency scale, ranging from 1 *(never) *to 5 *(always)*.

### Health measures

*Concentration problems *will be measured with three items derived from a semantic differential scale developed by Meijman [30]. The 5-point response scale have two extremes, for example *'No concentrating difficulties' *and *'Concentration difficulties'. Emotional exhaustion *will be measured by the well-validated Dutch version [31] of the Maslach Burnout Inventory [32]. The scale contained five items with a 7-point response scale ranging from 0 *(never) *to 6 *(always, daily)*. An example item is: "I feel emotionally drained from my work".

*Physical complaints *refer to neck, shoulder and back problems in the last six months and will be measured with three items derived from a scale developed by Hildebrandt and Douwes [33]. The possible responses are 1 *(no)*, 2 *(sometimes)*, and 3 *(yes)*.

### Well-being measures

*Job satisfaction & Work motivation *will be measured by scales developed by De Jonge [34]. Job satisfaction can be considered as a unidimensional and general construct, resulting from positive and negative work experiences. It will me measured with one item i.e. "I am satisfied with my present job". Work motivation is the extent to which the work is stimulating, interesting, and challenging and will be measured with five items. For example, "My work is very interesting". All items are measured on a 5-point Likert scale, ranging from 1 *(strongly disagree) *to 5 *(strongly agree)*.

### Performance measures

*Active learning *refers to the degree employees are enabled and stimulated to acquire new knowledge and skills, and to solve problems at their job. This scale [35] consists of four items that can be scored on a 4-point frequency scale, ranging from 1 (*(almost) never*) to 4 (*(nearly) always*). For example, "At work, I am challenged by new problems".

*Employee creativity *can be defined as the generation of novel and useful ideas by employees. This work-related construct will be assessed by a 7-item scale originally developed by George and Zhou [36], and translated/back-translated in a well-validated Dutch version [37]. The scale can be scored on a 5-point rating scale ranging from 1 (*never*) to 5 (*always*). For example "Comes up with new and practical ideas to improve performance".

### Development of workplace interventions

**Figure 2:**
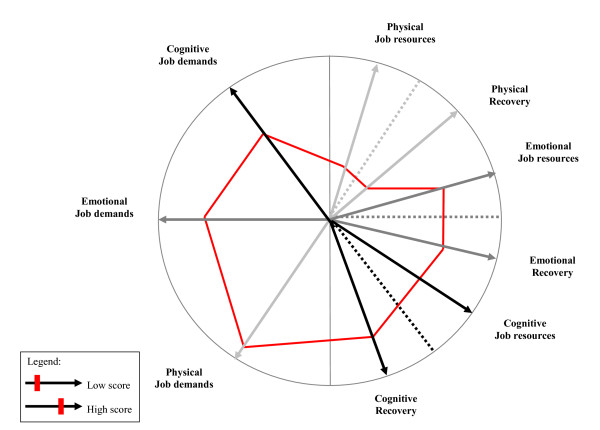
**Example of a risk-profile**.

**Figure 3:**
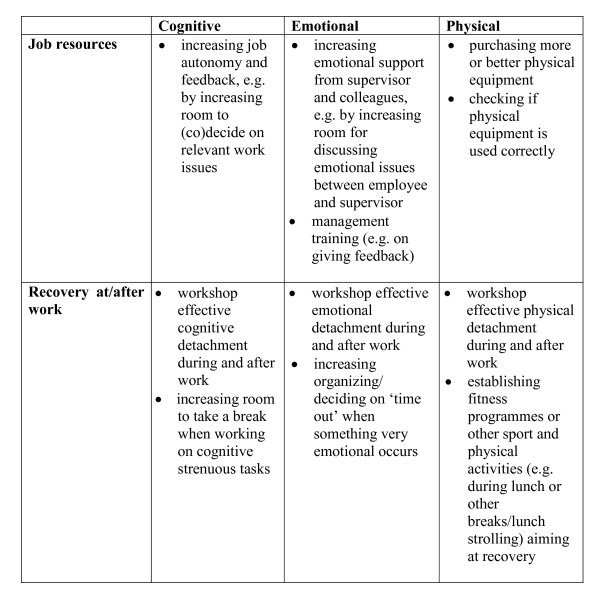
**Examples of work-oriented interventions based upon different kinds of job resources and recovery at/after work**.

Based on the results of the base-line survey the researchers will develop risk-profiles for each ward, which report the specific job demands, job resources, and recovery opportunities of the wards involved. Figure 2 presents an example of a risk-profile. It shows that particularly physical job resources and physical recovery are rather low to counteract the amount of physical job demands. The risk-profiles will be the starting-point for the researchers to generate ideas for workplace interventions that could be implemented to optimize job resources and recovery opportunities on the intervention wards. For developing and implementing interventions, a Participatory Action Research (PAR) approach will be used [38]. The philosophy behind PAR is that interventions designed to improve job-related outcomes cannot take place without the participation and experience of the subjects under study. The effectiveness of PAR in intervention research has been demonstrated empirically [39-41]. How will PAR be used in the present study? The results of the base-line survey together with the ideas for possible interventions from the researchers, will be discussed in small groups of employees from the experimental wards (i.e. intervention mapping). After consensus about interventions with the highest priority, employees will be made self-responsible for the implementation of (a part of) the interventions. During the process of developing and implementing interventions the researchers will be supported by an external consultant. How will the interventions look like? Figure 2 showed for example that the availability of physical job resources and physical recovery opportunities for this ward are rather low to counteract the relatively high physical job demands. To enlarge the amount of physical resources it can be important to check if there is sufficient adequate technical equipment to accomplish physically strenuous tasks and if this equipment is used correctly by the employees. Physical recovery at/after work can for example be improved by establishing fitness programmes or other sport and physical activities. To conclude, the ward-profiles will serve as the basis for the development of tailored ward-directed interventions. Figure 3 showed examples of workplace interventions based upon different kinds of job resources and recovery at/after work. All the interventions that will be implemented are primarily work-oriented rather than worker-oriented.

### Evaluating the effects of workplace interventions

**Figure 4:**
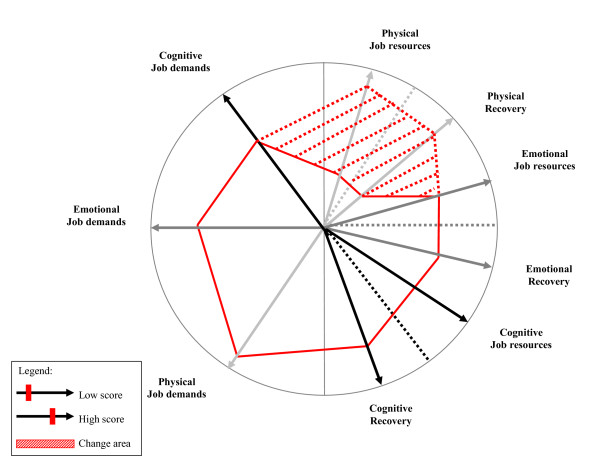
**Example of a change-profile**.

After the implementation of workplace interventions and the first follow-up survey, a change-profile will we developed for each ward. The change-profile from a ward is exactly the same as the risk-profile but contains also the scores on specific job demands, job resources, and recovery opportunities from the follow-up survey. Figure 4 shows an example of a change-profile, indicating a positive effect on physical job resources and physical recovery. Because job demands are difficult to change, these scores are almost equal as the scores from the base-line survey. However, the amount of physical job resources and physical recovery opportunities for this ward are increased, so the employees will be better able to counteract their relatively high physical job demands. The increase in specific job resources and recovery opportunities is called the change-area. The change-profile shows if an intervention is successful in increasing specific types of job resources and recovery opportunities. However, to determine the effect on an outcome measure (e.g. physical complaints), the average score of the first follow-up survey will be compared with the score from the base-line survey. To investigate the enduring effects of the interventions, change-profiles will be developed again after the second follow-up survey.

### Process evaluation

It is impossible to control all external influences on performance, well-being and health from the implementation of the interventions until the follow-up measures. However, some actions will be taken to get insight into factors that possibly affect the results. Therefore, a process evaluation will be carried out [42]. The aim of this evaluation is to map factors that either stimulated or hindered successful implementation as well as the effectiveness of the interventions. The managers of the involved wards will be asked to note important changes and events on their wards in a logbook. Both after the first follow-up survey an the second follow-up survey, in-depth interviews will be held with the managers and nursing staff of both the intervention and comparison wards to interpret possibly changes in performance, well-being, and health on their ward. It will also be checked if employees on the intervention wards really perceived interventions. At the last page of both the follow-up surveys, they will be asked to list actions (since the previous survey) that were taken to improve health, well-being and performance on their ward. The same will be done with employees of the comparison wards. Consequently, it can be checked if managers of the comparison wards implemented interventions on their own initiative. In general, we will follow the intervention evaluation criteria as depicted by Scharf et al. [28] as much as possible.

### Procedure

At the base-line measure, each survey will be provided with a random chosen identification number corresponding to the employee. This number will be retained and used for the follow-up measures. The identification number is only available for the researchers and will be only used for analysis purposes. After completing, the employees can return the survey to the central researcher with a return envelope.

### Statistical analysis

#### Sample size calculation

Sample size calculation is based on emotional exhaustion, measured by the Dutch version [31] of the Maslach Burnout Inventory [32]. This measure is chosen because of the availability of norm scores for (certified) nursing assistant, which is the most occurring grade on both the intervention and comparison wards. The score ranges between 1 and 7, with an average score of M = 1.74 and a standard deviation of SD = 94. Setting alpha at 0.05, beta as 0.10 and Δ = .47 (half a standard deviation as a clinically minimal relevant difference [43] results in a required N = 120 (N = 60 for the experimental group and N = 60 for the comparison group) [44]. However, eight wards (N = 300) were interested to participate in the research project. Taking drop-outs into account, this sample is expected to be large enough to detect significant effects.

#### Drop-outs

Drop-outs will be documented thoroughly and included in the data-analysis to the point of drop-out. Analysis according to the guidelines by Goodman and Blum [45] will be conducted to assess any attrition effects. For instance, mean differences on measures from the base-line survey between those who responded and did not respond to the follow-up surveys.

#### Data-analysis

Cross-sectional, baseline, relations between specific types of job demands, job resources, recovery and job-related outcomes will be tested with hierarchical regression analysis (SPSS for Windows) and structural equation modeling (LISREL for Windows). In order to analyze causal associations within the two different waves, structural equation modeling (LISREL for Windows) will be used, as this technique is more useful to 'prove' causation (i.e. to rule out alternative, causal, assumptions). To evaluate the results of the interventions after the follow-up measures, multilevel repeated measures analysis will be performed using MLwiN. This technique has several advantages compared to repeated measures MANOVA, like the inclusion of cases with incomplete data and less restrictive missing data assumptions.

#### Ethical considerations

Given the non-intrusive nature of the research, ethical approval seems to be not required. Nevertheless, we submitted the research proposal to the Medical Ethics Committee of the azM and Maastricht University, and she gave a positive advise. In addition, the research plan was presented to the union boards of the participating nursing homes and they gave their consent as well. Employees participate in the research project on a voluntary basis and confidentiality is guaranteed.

## Discussion

Although the literature showed that the availability of sufficient job resources and recovery opportunities have a positive influence on several job related outcomes, it is still unclear how job resources and recovery opportunities can be translated into effective workplace interventions aiming to improve employee health, well-being, and performance-related outcomes. The current research project will overcome this deadlock in research. It will be investigated how workplace interventions can be tailored to optimize job resources and recovery opportunities, in order to counteract job demands, and what the effects of these interventions will be on health, well-being, and performance of nursing home employees.

According to Kompier and Kristensen [19], a systematic analysis of risk factors is often lacking in stress intervention research. Hence, a strength of the present study is the adequate diagnosis of risk factors conform the principles of the Demand-Induced Strain Compensation (DISC) Model [6,20]. Because only a few studies assessed the impact of work-oriented interventions [26], investigating the effects of these interventions on health, well-being, and performance is a second strength of the current study. A third strength of the research project is the use of a Participatory Action Research (PAR) approach [38], through which the commitment of employees during the process of developing and implementing interventions will be stimulated. A final strength of this study is the comparison of different interventions on respective, similar, outcomes. According to Richardson and Rothstein [26], this is an important contribution to both theory and practice.

Besides the obvious strengths of the present study, there are also some limitations. First, the design of the study is not a true experimental design. The wards participating in the present research project are existing organization units. Due to ethical and practical drawbacks, randomization is difficult to realize. However, the experimental- and comparison wards were selected in pairs so each intervention department has a similar comparison department. According to Ovretweit [46] 'Traditional experimental evaluation design is not well suited to investigating social systems or the complex way in which interventions work with subjects or her environment' (p99). Secondly, It is impossible to control all external influences on performance, well-being and health from the implementation of the interventions until the follow-up measures. To overcome this problem a thoroughly process evaluation will be conducted to detect and interpret external influences.

Although the limitations, the design of the present study is a feasible method to assess the effect of optimizing job demands, job resources, and recovery on health, well-being and performance related outcomes. The study is in progress. The baseline paper-and-pencil survey was conducted in the Spring of 2009. Interventions will be developed and scheduled to be implemented within due course. Follow up measurements are planned in 2010 and 2011, respectively. Dissemination of results is planned for the end of 2011.

## Competing interests

The authors declare that they have no competing interests.

## Authors' contributions

ES, JDJ, and JPHH were involved in the design of the study. ES drafted the manuscript and performed the statistical analysis. JDJ and JPHH critically reviewed the manuscript. All authors read and approved the final manuscript.

## Pre-publication history

The pre-publication history for this paper can be accessed here:

http://www.biomedcentral.com/1471-2458/10/293/prepub
